# Exploring the clinical significance of anti-acetylcholine receptor antibody titers, changes, and change rates in Myasthenia Gravis

**DOI:** 10.3389/fneur.2024.1506845

**Published:** 2025-01-15

**Authors:** Lijun Luo, Xinyi Zhu, Chunbei Wen, Yifan Guo, Jie Yang, Dongsheng Wei, Ping Yu, Mei Wan

**Affiliations:** ^1^Department of Neurology, Wuhan No. 1 Hospital, Wuhan, China; ^2^The First Clinical Medical Institute, Hubei University of Chinese Medicine, Wuhan, China

**Keywords:** Myasthenia Gravis, acetylcholine receptor antibody titers, Myasthenia Gravis disease severity score, Myasthenia Gravis activities of daily living scale score, biomarker

## Abstract

**Introduction/Aims:**

Myasthenia Gravis (MG) is a common neuromuscular junction disorder that is primarily mediated by anti-acetylcholine receptor antibodies (AChR-Ab). However, using AChR-Ab titers to predict MG severity and improvement remains controversial. This study aims to explore the relationship between AChR-Ab titers and AChR-Ab rate of change (RR-AChR-Ab, %) and MG scores.

**Methods:**

We used a prospective study approach, and included 62 patients with generalized MG (GMG) who were positive for AChR-Ab. We measured AChR-Ab titers, MGFA-QMGS, and MG-ADL scores at baseline (before treatment) and at 3 and 6 months into treatment. Pearson and Spearman correlation analyses were used to study the relationships between changes in AChR-Ab titers, rates of change, and MG scores.

**Results:**

(1) At baseline, there was no correlation between AChR-Ab titers and age, duration of illness, gender, MGFA classification, or presence of thymic abnormalities. (2) The trend of decreasing AChR-Ab titers matched the trend of reduced QMGS and ADL scores. (3) Six months into treatment,there was a correlation between AChR-Ab titer changes and changes in ADL scores. (4) Three months into treatment, RR-AChRAb showed a correlation with the rate of change in ADL at the same time point.

**Conclusion:**

We found the trend of decreased AChR-Ab titers after standardized treatment that was consistent with reductions in QMGS and ADL scores. Additionally, the rate of change in AChR-Ab titers at 3 months and the change in AChR-Ab titers at 6 months into treatment did reflect improvements in activities of daily living for MG patients.

## 1 Introduction

Myasthenia Gravis (MG) is an autoimmune disorder characterized by impaired neuromuscular junction transmission because of pathogenic autoantibodies that target the postsynaptic membrane (1). Approximately 85% of patients with generalized MG (GMG) are positive for acetylcholine receptor antibodies (AChR-Ab), and are clinically classified as having the AChR-MG subtype (2) The exact pathogenic mechanisms of MG are not fully understood, but previous studies have shown that AChR-Abs can cause disease via three main mechanisms: (1) They initially bind to the acetylcholine receptors (AChR) located at the neuromuscular junction, forming antigen–antibody complexes. Subsequently, this interaction triggers the complement system, resulting in the formation of membrane attack complexes. These complexes have the potential to disrupt the muscle membrane at the neuromuscular junction, thereby impairing neuromuscular transmission and leading to compromised motor function; (2) through antigenic modulation, they can accelerate the degradation and internalization of acetylcholine receptor (AChR), thus reducing the amount of active AChR on the end plate; (3) They can block acetylcholine-AChR interactions by binding to the same site or close to it, thus reducing ion channel traffic and disrupting neuromuscular transmission (3–6). Among them, complement involving damage the postsynaptic membrane has a significant impact on AChR-MG (7, 8). Fluctuating muscle weakness, electrophysiological findings, and serological antibodies can all be used to diagnose MG, with a serum AChR-Ab titer ≥0.50 nmol/L defined as positive for AChR-MG (9). Although AChR-Ab titers may help diagnose and serologically classify MG, the relationship between AChR-Ab titers and disease severity, as well as possible correlations between changes in AChR-Ab titers and changes in MG symptom scores, remain controversial (10–14).

Numerous studies have shown that serum AChR-Ab titers do not significantly correlate with clinical MG severity (4, 15–17), but other studies have shown that a correlation exists, with patients with more severe symptoms tending to have higher antibody titers (10, 18–21). Specifically, one study indicated that an AChR-Ab titer >23.11 nmol/L could predict if a patient was experiencing an acute MG exacerbation (22). As treatment progresses, antibody testing can influence therapeutic decisions. Rising antibody levels are considered a sign of disease progression, whereas stable or declining levels may indicate disease stability (20). Additionally, among patients with ocular MG (OMG), those with higher AChR-Ab titers have a greater risk of progression to GMG (23). Numerous studies have investigated potential correlations between AChR-Ab titers and MG severity. However, most of those studies were retrospective, and the few prospective clinical studies that have been conducted did not employ precise subgroup classifications or use standardized immunotherapy to control for confounding factors (11). Thus, we conducted a prospective clinical study which measured AChR-Ab titers at baseline, at 3 month into treatment, and at 6 months into treatment. We also concurrently assessed scales of subjective MG symptom severity (including the MG Foundation of America (MGFA) disease severity scores(MGFA-QMGS) (24) and MG activities of daily living scale(MG-ADL) (25, 26))to analyze the relationship between the titers of AChR-Ab, their changes, and change rates, and the MG scale scores, their changes, and change rates. Through this design, we aimed to explore the clinical significance of AChR-Ab in the assessment of MG.

## 2 Methods

### 2.1 Study design and data source

We enrolled patients with MG who were admitted to the Department of Neurology at Wuhan No. 1 Hospital between August 2021 and August 2023. The inclusion criteria were as follows: Patients diagnosed with AChR-Ab positive MG who were also—(1) aged 18 to 85 years, with an MGFA classification of IIa-IVb; (2) naïve to non-steroidal immunosuppressants prior to the study, or who had used them but had discontinued use for over 6 months, or were untreated, or were newly diagnosed patients; (3) able to understand and sign informed consent forms. Exclusion criteria were as follows: (1) concurrent malignancy (except thymoma); (2) severe active hepatitis B or C; (3) active tuberculosis; (4) severe liver or kidney dysfunction, multiple organ failure; (5) severe allergy or infection; (6) pregnant and lactating women.

Patient demographics (age, gender), disease duration, MGFA classification, thymus CT scans, QMGS, ADL scores, and AChR-Ab titers were recorded. AChR-Ab (binding antibody) testing was performed in the Guangzhou OmnoMed medical laboratory using the Radioimmunoassay (RIA) method to analyze serum samples, with AChR-Ab titers ≥0.5 nmol/L considered positive.

All enrolled patients received standardized medical treatment, symptomatic treatment (Pyridostigmine Bromide tablets) and immunotherapy (corticosteroids + tacrolimus or AZA), with medication doses adjusted based on treatment responses. MGFA-QMGS and MG-ADL assessments, as well as AChR-Ab titer measurements, were performed at baseline, 3 months, and 6 months into treatment. Baseline results were used as the initial data, and changes and rates of change in AChR-Ab titers were calculated from baseline to after treatment.

Changes and rates of change in AChR-Ab titers, QMGS, and ADL scores were calculated using *Δ* values (Δ = baseline value - post-treatment initiation value) and RR values (RR = (baseline value - post-treatment initiation value) / baseline value * 100). Changes at 3 months were denoted as Δ1, and at 6 months as Δ2; rates of change at 3 months were marked as RR1, and rates of change at 6 months were marked as RR2.

### 2.2 Ethical approval

All patients provided written informed consent prior to inclusion in the study, which was also approved by the local ethics committee (Ethics Committee of Wuhan No. 1 Hospital, approval no. 2021–34; approved on September 9, 2021).

### 2.3 Statistical analysis

Statistical analysis was performed using IBM SPSS Statistics (version 25), and all graphs were created using GraphPad Prism version 9. Normality tests were conducted for all continuous variables. Variables conforming to a normal distribution were expressed as means ± standard deviations (SD), and those not conforming were expressed using medians and interquartile ranges (IQR). Percentages were used when necessary. To determine if there are differences in AChR-Ab titers among different genders, disease durations, MGFA classifications, and the presence or absence of thymic abnormalities at baseline, independent sample t-tests were conducted for comparisons between two groups, and one-way ANOVA was used for comparisons involving more than two groups. The Friedman test was utilized to assess within-subject variations in AChR-Ab titers, QMGS, and ADL scores at distinct time points post-treatment. Pearson and Spearman correlation analyses were employed to assess the relationships between AChR-Ab titers, changes, and rates of change with QMGS and ADL scores, including their changes and rates of change at baseline, as well as at 3- and 6-months into treatment. *p*-values <0.05 were considered statistically significant.

## 3 Results

### 3.1 Baseline characteristics of patients

Our study included 62 patients with generalized MG who were positive for AChR-Ab. One patient had an abnormally high AChR-Ab titer at enrollment, which was considered an outlier and thus excluded from statistical analysis. The median age was 54 years (interquartile range: 40.5–67); 42.62% of our patients (26 patients) were male, and 57.38% (35 patients) were female. Disease duration was less than 1 year for 50.82% of our sample (31 patients), 1–5 years for 32.79% (20 patients), and more than 5 years for 16.39% (10 patients). According to MGFA classifications, 42.62% of patients (26 patients) were class II, 55.74% (34 patients) were class III, 1.64% (1 patients) were class IV. Thymic abnormalities were present in 50.82% (31 patients) of the cases, including 10 with thymoma, 7 post-thymectomy, 13 with thymic atrophy, and 1 with thymic hyperplasia; 49.18% (30 patients) had no significant thymic abnormalities.

### 3.2 Correlation analyses of AChR-ab titer and age, gender, disease duration, MGFA classification, and thymic abnormalities at baseline

At baseline, No statistically significant differences were observed in AChR-Ab titers across different genders, durations of disease, MGFA classifications (since there was only one IV case, analysis was performed by grouping II, III + IV) or presence of thymic abnormalities (Table 1). Pearson and Spearman correlation analyses were conducted to assess the relationship between AChR-Ab titers and various demographic and clinical variables. The results indicated no significant correlation between AChR-Ab titers and age (Spearman’s *ρ* = 0.119, *p* = 0.362), as well as no significant correlations with gender, durations of disease, MGFA classification or presence of thymic abnormalities (*ρ* = −0.162, *p* = 0.213; ρ = −0.058, *p* = 0.655; ρ = −0.048, *p* = 0.711; ρ = −0.060, *p* = 0.648, respectively).

**Table 1:** Comparison of AChR-Ab titers in different sex, disease duration, MGFA classification and with or without thymic abnormalities.

	AChR-Ab Titers (nmol/L)	*F*/*t* value	*p* value
Sex		1.434	0.157
Male (*n* = 26)	(10.492 ± 4.238)		
Female (*n* = 35)	(8.814 ± 4.714)		
Disease duration		0.877	0.421
Less than 1 year (*n* = 31)	(9.723 ± 4.560)		
1–5 years (*n* = 20)	(10.084 ± 4.430)		
More than 5 years (*n* = 10)	(7.818 ± 4.868)		
MGFA classifications		1.135	0.261
Class II (*n* = 26)	(10.296 ± 4.909)		
Class III + class IV (*n* = 35)	(8.960 ± 4.262)		
Thymic abnormalities		0.693	0.491
Yes (*n* = 31)	(9.130 ± 4.531)		
No (*n* = 30)	(9.942 ± 4.627)		

### 3.3 Relationship between AChR-ab titers, QMGS, ADL scores, and follow-up time

**Figure 1:**
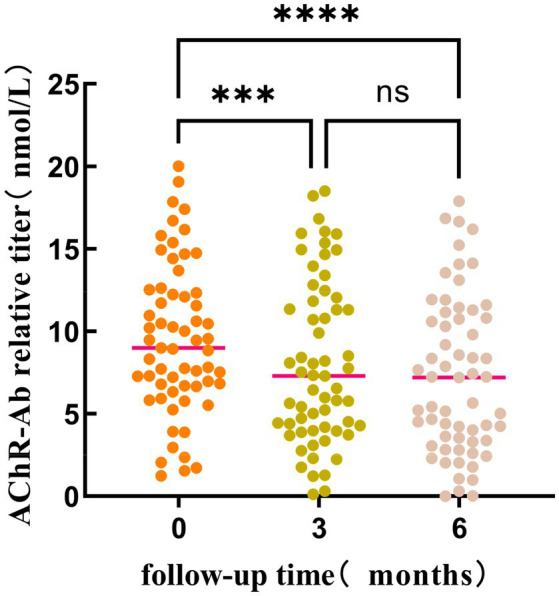
The differences in AChR-Ab titers at different time points (baseline, 3 months into treatment, and 6 months into treatment) were statistically significant (*p* < 0.001). Post-hoc analysis with Bonferroni correction revealed that the AChR-Ab titers at baseline were significantly higher than those at both 3 months (*p* < 0.001) and 6 months into treatment. However, the difference in AChR-Ab titers after 3 and 6 months into treatment was not significant after adjustment for multiple comparisons (*p* = 0.129). ***, ****p < 0.001; ns: *p* = 0.129.

**Figure 2:**
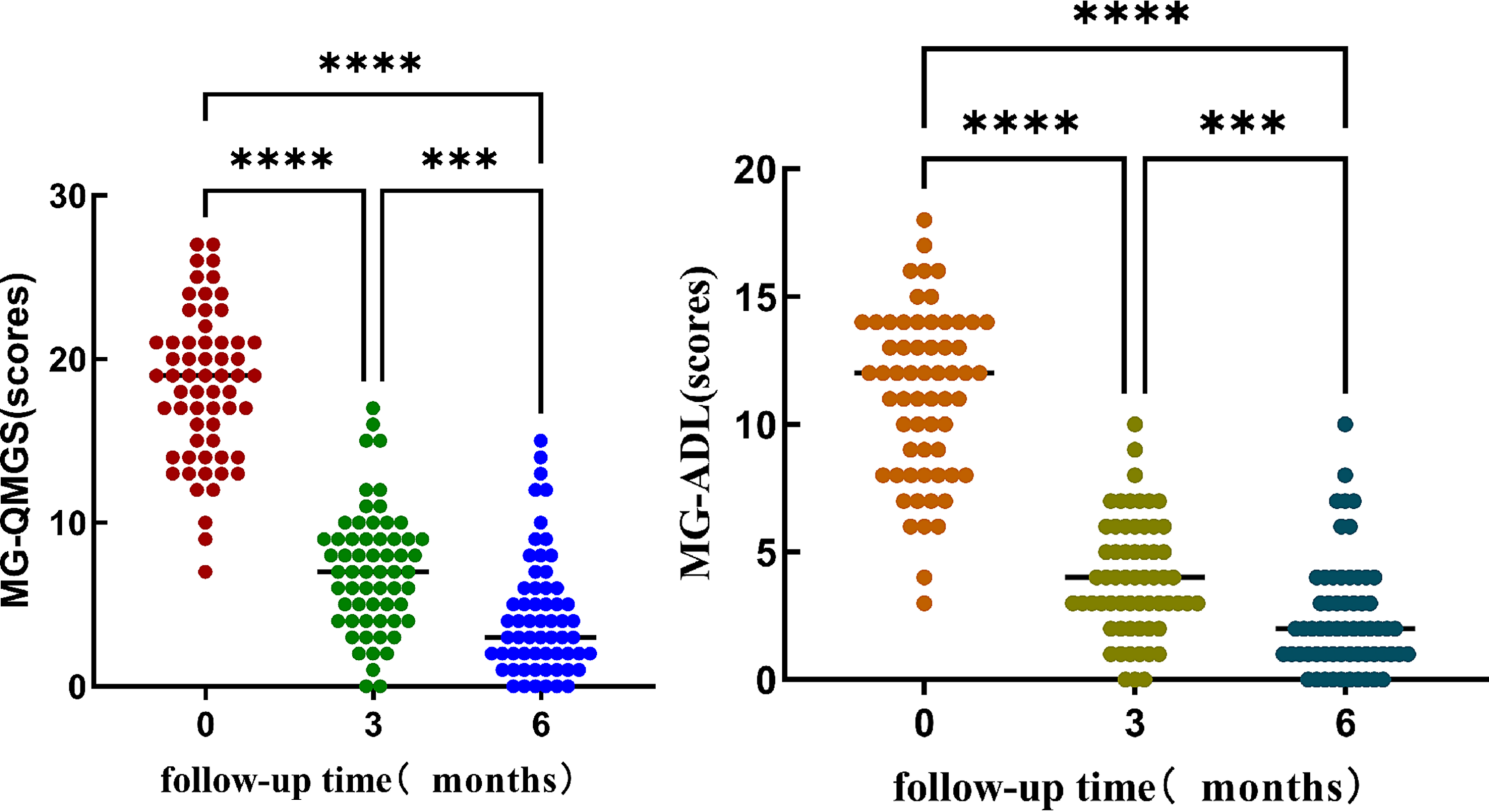
The significant differences in QMGS and ADL scores at different time points (baseline, 3 months into treatment, and 6 months into treatment). Post-hoc multiple comparisons with Bonferroni correction indicated that both QMGS and ADL scores at baseline were significantly higher than those at 3 and 6 months into treatment (*p* < 0.001). Additionally, the QMGS and ADL scores at 3 months into treatment were significantly higher than at 6 months into treatment (*p* < 0.001).

Friedman test was utilized to assess the trends in AChR-Ab titers, QMGS, and ADL scores across different time points. Where the Friedman test indicated statistically significant differences, post-hoc pairwise comparisons with Bonferroni correction were conducted. The results revealed that with the extension of standardized treatment, there was a decrease in AChR-Ab titers, QMGS, and ADL scores. Significant differences in AChR-Ab titers were observed between baseline and at 3 months into treatment (*p* = 0.001) and between baseline and at 6 months into treatment (*p* < 0.001). However, the difference in AChR-Ab titers after 3 and 6 months into treatment was not significant after Bonferroni correction for multiple comparisons (*p* = 0.129), suggesting that the primary decline in antibody titers occurred within the first 3 months of treatment (Figures 1, 2).

To assess the correlation of AChR-Ab titers, QMGS, and ADL scores over time, we employed Spearman’s rank correlation coefficient analysis on the longitudinal data at three time points. A significant correlation was observed between the decreasing trend of AChR-Ab titers and the reduction in QMGS scores (*ρ* = 0.187, *p* = 0.012) and ADL scores (*ρ* = 0.241, *p* < 0.05). Additionally, a high correlation was found between the reduction trends of QMGS and ADL scores (*ρ* = 0.902, *p* < 0.001). These results suggest that the downward trend in AChR-Ab titers is consistent with the improvement in QMGS and ADL scores, with a stronger association with ADL scores.

### 3.4 Correlation analysis of AChR-ab titers and QMGS and ADL scores at baseline, 3 months, and 6 months into treatment

At baseline, Pearson correlation analysis revealed no significant relationship between AChR-Ab titers and QMGS or ADL scores (*r* = 0.191, *p* = 0.141; *r* = 0.218, *p* = 0.091). Spearman correlation analyses conducted at 3 months and 6 months into treatment also showed no significant correlations between AChR-Ab titers and QMGS or ADL scores at these time points (*r* = 0.002, *p* = 0.989; *r* = 0.134, *p* = 0.302 for 3 months and *r* = 0.002, *p* = 0.989; *r* = 0.090, *p* = 0.491 for 6 months).

### 3.5 Correlation analysis of ∆AChR-ab titer and ∆QMGS and ∆ADL scores at 3- and 6-months into treatment

**Figure 3:**
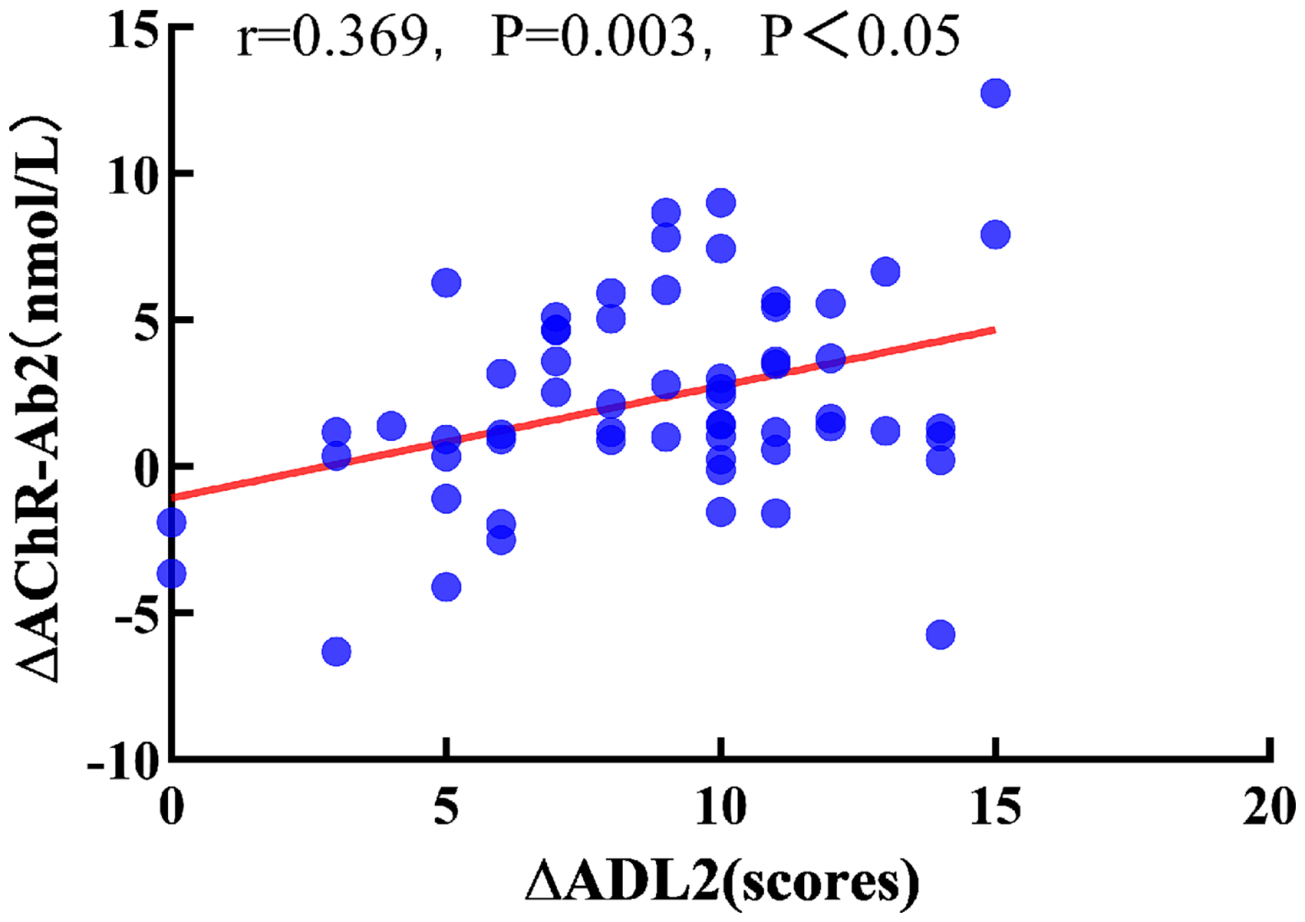
Relationship between ∆ADL2 and ∆AChR-Ab2. Pearson correlation coefficient, red dashed line is the linear regression line.

Pearson correlation analysis showed that, after 3 months of treatment, ∆AChR-Ab1 had no significant correlation with ∆QMGS1 or ∆ADL (*r* = 0.163, *p* = 0.210; *r* = 0.255, *p* = 0.047, respectively). After 6 months of treatment, ∆AChR-Ab2 had no significant correlation with ∆QMGS2 (*r* = 0.225, *p* = 0.081), but was moderately correlated with ∆ADL2 (*r* = 0.369, *p* = 0.003, *p* < 0.05; Figure 3).

### 3.6 Correlation analysis: RR-AChR-ab titer and RR-QMGS,and RR-ADL scores 3- and 6-months into Treatment3

**Figure 4:**
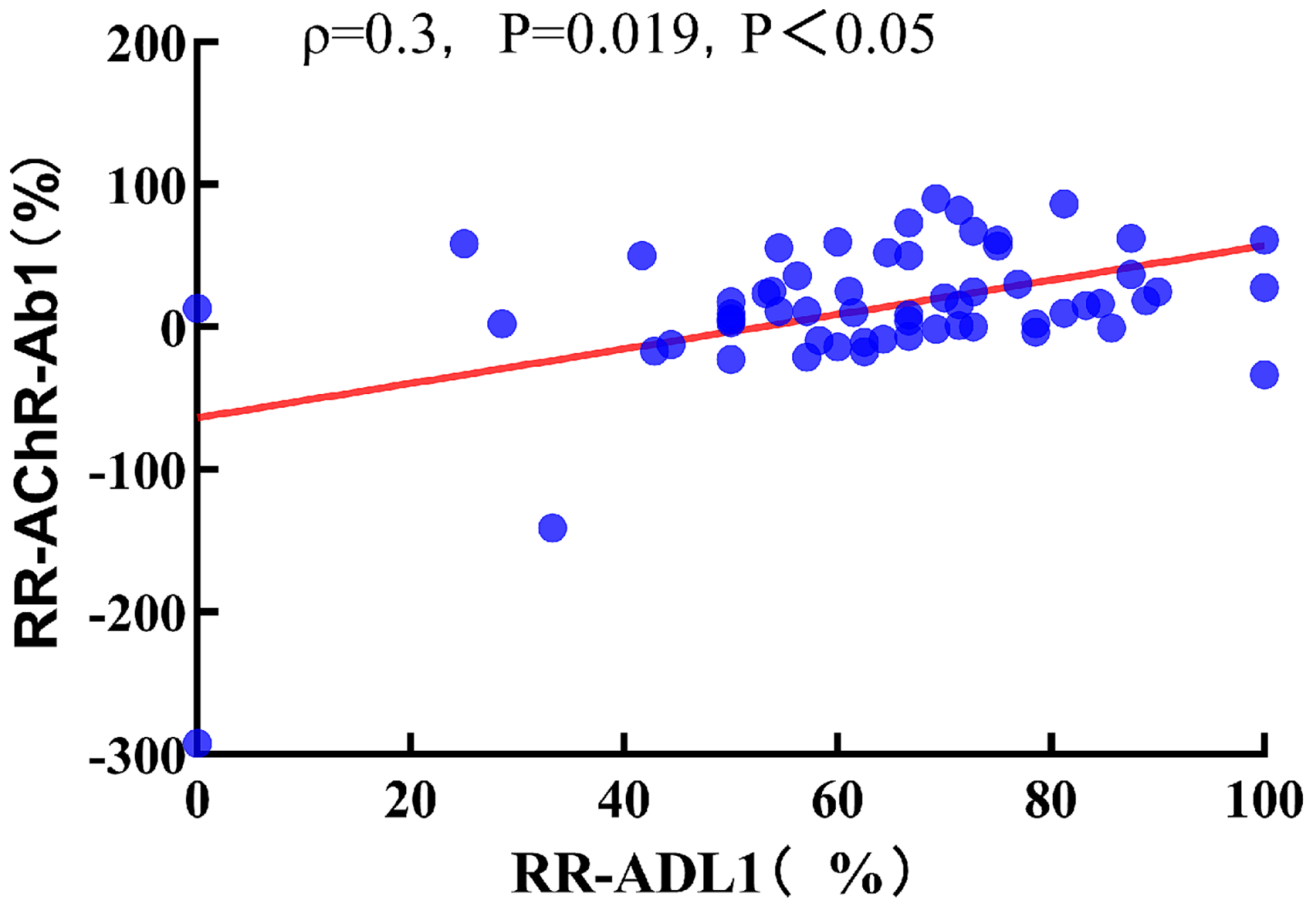
Relationship between RR-ADL1 and RR-AChR-Ab1. Spearman correlation coefficient, red dashed line is the linear regression line.

Spearman correlation analysis indicated that the RR-AChR-Ab1 at 3 months was not correlated with RR-QMGS1 (*ρ* = 0.101, *p* = 0.458). However, it was significantly correlated with RR-ADL1(ρ = 0.3, *p* = 0.019, *p* < 0.05; Figure 4). RR-AChR-Ab2 titers at 6 months were not significantly correlated with RR-QMGS2, or RR-ADL2 (*ρ* = 0.057, *p* = 0.660; *ρ* = 0.176, *p* = 0.176, respectively).

## 4 Discussion

Our study results indicate no differences in AChR-Ab titers across different patient subgroups based on age, gender, disease duration, or thymic abnormalities, which is consistent with prior findings by Aguirre et al., who also reported no differences in AChR-Ab titers in patients with different genders, thymic abnormalities, and histories of thymectomy (20). However, Monte et al. found that titers increased with age, indicating a correlation between antibody titers and age (27). Additionally, work by Somnier et al. suggested that antibody titers correlated with gender and thymic abnormalities, with thymic hyperplasia associated with higher AChR-Ab titers (18). Our results diverge from these studies, which may be due to our relatively small sample size, emphasizing the need for further research with larger sample sizes and multicenter clinical observations.

Lindstrom conducted a cross-sectional study (15) which showed no correlations between AChR-Ab titers and Osserman classification or pharmacological classification, a result also supported by Aurangzeb’s work (16). However, these retrospective cross-sectional clinical studies could not control for the influence of numerous other variables. Additionally, the Osserman classification system cannot precisely assess symptom severity, potentially introducing analytic errors. In contrast, several studies using the MGFA classification (28) have demonstrated correlations between AChR-Ab titers and MGFA classifications (19, 29, 30), but our findings indicate no correlation between AChR-Ab titers and MGFA classifications, possibly due to the small sample size and relatively high concentration of patients in the III category (55.74%), which could have impacted our outcomes.

Numerous studies continue have shown inconsistencies between serum AChR-Ab titers and the clinical severity of MG (4, 15–17, 31). Thus, it is not advocated to use the titers of AChR-Ab to assess the severity or clinical improvement of the patients. Instead, tools like MG-ADL and MGFA-QMGS are recommended for evaluating severity and symptom improvement (32–34). Our study findings, which show no significant correlation between AChR-Ab titers and QMGS or ADL scores at baseline, 3 months, or 6 months into treatment, align with this perspective and with previous research findings. The lack of correlation between AChR-Ab titers and clinical severity might be explained by several factors. First, AChR-Ab can be categorized into three subtypes: binding, blocking, and modulating antibodies. Studies have found that patients who are positive for both binding and blocking antibodies exhibit greater clinical severity than those who are positive for only one (35). However, the RIA primarily measures binding antibody titers (21), and could potentially miss other types of antibodies that may affect disease severity. Second, AChR-Ab is polyclonal, and capable of recognizing multiple antigen epitopes, with different epitopes causing varying degrees of pathogenicity. A single MG autoantibody clone can mediate multiple pathogenic mechanisms (36), and the synergistic interaction among different epitope-specific antibodies can enhance pathogenic effects (37). Thus, the measured antibody titers may not correlate well with clinical severity due to these complex interactions and variations in pathogenicity.

The debate over the clinical significance of continuously monitoring AChR-Ab titers and using changes in antibody levels to assess patient responses to treatment persists [10–14, 38, 39]. On one hand, some studies indicate that titer changes do not correlate with improvements in disease severity (38). For example, Lee and others have argued that the utility of AChR-Ab titers for tracking MG clinical progression is limited, and do not recommend repeated measurements (39). On the other hand, numerous studies have suggested a relationship between changes in AChR-Ab titers and improvements in clinical symptoms (40–42). A long-term follow-up study on AChR-Ab titers and disease severity (43) found no direct connection between AChR-Ab levels and clinical scores, but noted a consistent trend between decreases in antibody titers and improvements in QMGS. A different study introduced the concept of the rate of change in AChR-Ab levels (RR-AChR-Ab, %/d) (44), and found a strong correlation between declines in the rate of RR-AChR-Ab and changes in ADL scores, and suggested retesting AChR-Ab titers within 100 days after starting immunosuppressive treatment. Similarly, we found that, after initiating standardized treatment in MG patients, decreases in AChR-Ab titers coincided with decreases in QMGS and ADL scores, indicating that the decrease in AChR-Ab antibody titers correlates with improvements in clinical symptoms and enhanced self-care capabilities in patients. Moreover, the primary decrease in AChR-Ab titers occurred within the first 3 months of treatment. After the first 3 months, antibody changes were less notable, possibly due to the stabilization of patient conditions after initiation of treatment. Additionally, we found a correlation between the RR-AChR-Ab1 (the rate of change in AChR-Ab titers) at 3 months and the RR-ADL1(the rate of change in ADL scores), as well as a correlation between the ΔAChR-Ab2 (the change in AChR-Ab titers) at 6 months and the ΔADL2 (the change in ADL scores). Chou et al. (45) also discovered that AChR-Ab levels could predict long-term outcomes. Consequently, monitoring antibody titers in patients following the initiation of immunosuppressive therapy, along with calculating the changes and rates of change, can be used to demonstrate the efficacy or failure of the immunosuppressive treatment, and has certain guiding significance for the progression and prognosis of the disease.

In addition to AChR-Ab titers, QMGS, and ADL scores, research into potential biomarkers of MG treatment efficacy has suggested that levels of the AChR1 subunit protein (46) and circulating micro RNAs (miRNA) (47) could potentially serve as indicators of disease activity in AChR-Ab positive MG patients. Additionally, peripheral blood hsa-circRNA5333-4 levels are strongly correlated with QMGS (48). These novel biomarkers still need further clinical validation.

In conclusion, although AChR-Ab titers may not serve as markers of disease severity, the trend of decreased AChR-Ab titers after treatment initiation consistently aligns with reductions in QMGS and ADL scores. Additionally, the rate of change in AChR-Ab titers correlates with changes in ADL scores after 3 months of treatment; at 6 months, the change in AChR-Ab titers correlates with changes in ADL scores. Therefore, we recommend monitoring AChR-Ab titers at critical follow-up time points (such as 3 and 6 months after treatment initiation, or when there are changes in symptoms) in AChR-Ab positive MG patients undergoing standardized treatment, because AChR-Ab titers still hold clinical and prognostic value. To further understand the role of AChR-Ab titers as potential markers of MG severity, additional multicenter, large-scale, and long-term prospective clinical studies are needed.

## Data Availability

The raw data supporting the conclusions of this article will be made available by the authors, without undue reservation.
